# Visualization of 4D multimodal imaging data and its applications in radiotherapy planning

**DOI:** 10.1002/acm2.12209

**Published:** 2017-10-29

**Authors:** Matthias Schlachter, Tobias Fechter, Sonja Adebahr, Tanja Schimek‐Jasch, Ursula Nestle, Katja Bühler

**Affiliations:** ^1^ VRVis Research Center Vienna Austria; ^2^ Department of Radiation Oncology University Medical Center Freiburg Freiburg Germany; ^3^ German Cancer Consortium (DKTK), Partner Site Freiburg Heidelberg Germany

**Keywords:** 4D multimodal visualization, 4D‐PET/CT, radiotherapy planning, volume navigation

## Abstract

**Purpose:**

To explore the benefit of using 4D multimodal visualization and interaction techniques for defined radiotherapy planning tasks over a treatment planning system used in clinical routine (C‐TPS) without dedicated 4D visualization.

**Methods:**

We developed a 4D visualization system (4D‐VS) with dedicated rendering and fusion of 4D multimodal imaging data based on a list of requirements developed in collaboration with radiation oncologists. We conducted a user evaluation in which the benefits of our approach were evaluated in comparison to C‐TPS for three specific tasks: assessment of internal target volume (ITV) delineation, classification of tumor location in peripheral or central, and assessment of dose distribution. For all three tasks, we presented test cases for which we measured correctness, certainty, consistency followed by an additional survey regarding specific visualization features.

**Results:**

Lower quality of the test ITVs (ground truth quality was available) was more likely to be detected using 4D‐VS. ITV ratings were more consistent in 4D‐VS and the classification of tumor location had a higher accuracy. Overall evaluation of the survey indicates 4D‐VS provides better spatial comprehensibility and simplifies the tasks which were performed during testing.

**Conclusions:**

The use of 4D‐VS has improved the assessment of ITV delineations and classification of tumor location. The visualization features of 4D‐VS have been identified as helpful for the assessment of dose distribution during user testing.

## 1 INTRODUCTION

Modern radiation therapy aims at delivering high doses very precisely to a target volume with steep dose gradients to the surrounding organs at risk (OAR). Prerequisites therefore are very precise delineations. Although image‐guided radiation therapy allows for treatments with high precision, it is only as good as the accuracy with which the target is known.^1^ A high degree of uncertainty is associated with the delineation of the target volume^1^ and the traditional way to deal with these types of uncertainties is by extending delineations with an appropriate margin. For moving target, one commonly applied strategy comprises the generation of an internal target volume (ITV)^2^ from different time bins of 4D imaging data, for instance 4D‐CT. However, it remains challenging to efficiently navigate, visualize, and interpret these 4D imaging data.^3^ Due to limited time of physicians and lacking tools for dealing with 4D data efficiently, time effort is often reduced by using only the two extreme phases for target delineation.^4^ This neglect of large parts of the movement correlated data introduces another source of uncertainty, and might lead to inaccuracy in target volume delineation. Furthermore, as additional information of co‐registered functional imaging is increasingly employed in target volume delineation (e.g. 4D‐PET), the problem is aggravated, when these additional imaging data should be used in the planning process.

Visualization to efficiently use 4D multimodal imaging data is to the best of our knowledge not sufficiently implemented in currently available systems. Due to this unmet need, we developed a 4D multimodal visualization system (4D‐VS) that features fusion of 3D/4D multimodal image information, delineations of tumor and OARs as well as dose distribution data. A high emphasis was laid on interaction allowing for changing time bins, clipping volume information, segmentation and iso‐dose surfaces. In this article, we present a visualization system and its evaluation with respect to specific radiotherapy planning tasks. The rendering framework is based on a revised and extended list of requirements, which was presented in Ref. 5.

### 1.A Clinical requirements and tasks

Based on discussions with radiation oncologists, we developed a list of requirements to support radiotherapy planning tasks which incorporate 4D multimodality imaging. This includes visualization features which should be available early in the radiotherapy workflow when target and OARs are delineated, and in a later phase after the dose calculation was performed. Including 4D imaging data should make it especially suitable for cases with moving targets, for instance lung tumors, to ensure high accuracy delineations and coverage over the breathing cycle. Our visualization system is based on the following requirements:

1. Visualization and fusion of 4D (3D + t) multimodal data sets with support for changing time bins and data sets easily.
2. Joint visualization of segmentation data, such as ITV and OAR, and multimodal data sets.
3. Joint visualization of dose information (iso‐dose surfaces) and multimodal data/segmentation data.
4. Clipping and/or masking (using segmentation data) in the volume visualization.
5. Support of mixed resolution data sets without resampling and no preprocessing for volume fusion.
6. Interactive modification of parameters for clipping and visual appearance (e.g. fusion parameters).
7. Support for navigation from the volume visualization to the slice‐wise views.
8. Support for highlighting volume intersections.

Furthermore, we identified three clinically relevant tasks which form the basis to evaluate the visualization system:

T.1 Quality assessment of ITV contours
T.2 Classification of tumor location
T.3 Assessment of dose distribution.

Our tasks are motivated by patients who are scheduled for and/or treated by stereotactic body radiation therapy (SBRT). Task T.1, although not specific to SBRT, is very important when using SBRT due to the high doses involved. It will usually be performed simultaneously with the actual delineation task of target volumes. However, if the target is delineated using two extreme phases only, quality assessment for the remaining time bins is an equally relevant task. The classification of tumor location (T.2) is relevant to decide whether or not the patient should be treated with SBRT or receive conventional treatment. The assessment of dose distribution T.3 is also not specific to SBRT, but due the high doses involved, visualization techniques other than using the dose volume histograms (DVH) can be of interest in complicated cases, where the target is spatially close to an OAR.

### 1.B Related work

Visualization of multimodality data sets and the use of segmentation information for volume masking were presented in Ref. 6. Rendering multiple arbitrarily overlapping multiresolution volumes was covered by,^7^ and advanced support for clipping the volume visualization using mesh data was presented in Ref. 8. Specific work on PET/CT visualization with advanced functionality for fusion and clipping can be found in Refs. 9 and 10.

There have been efforts to bring visualization approaches like the aforementioned to frameworks such as the Visualization Toolkit (VTK).^11^ However, VTK still lacks multivolume rendering as reported by the visualization literature and extensions for multivolume visualization, for instance,^12^ have not found their way into the framework yet. Research platforms, such as 3D Slicer^13^ and the Medical Imaging Interaction Toolkit (MITK)^14^ which are tailored to medical applications often use VTK as basis for the visualization. They offer solutions to more specific clinical applications or workflows, but they also target data processing aspects and try not necessarily to improve the visualization. For example, SlicerRT^15^ is an extension to 3D Slicer with radiotherapy‐specific functionality, but it is more focused on data processing.

Commercial software products are used in clinical routine. These are for instance Mirada (Mirada Medical, UK), RayStation (RaySearch Laboratories AB, Stockholm, Sweden), MIM,^16^ Velocity^17^ and Oncentra MasterPlan (v4.3, Nucletron BV, Veenendaal, the Netherlands). But there is still a gap between what can be found in visualization literature and what has made its way into commercial products. To the best of our knowledge, none of the aforementioned products supports advanced visualization in 3D/4D as intended by our visualization system.

## 2 METHODS AND MATERIALS

The main focus of our visualization system is to improve radiotherapy planning‐related tasks by including multimodal volume visualization in an easy to use way. It is based on an in‐house developed multimodal rendering framework. Further interaction features are implemented alongside with the user interface within the MITK^18^ platform. Parameters which should be interactively modifiable by the user (requirement 6) have dedicated user interface elements implemented as MITK plugins. We refer to this as 4D‐VS. A video illustrating the main features (explained in the following) is available in the supporting information.

### 2.A Multimodal data description

**Figure 1:**
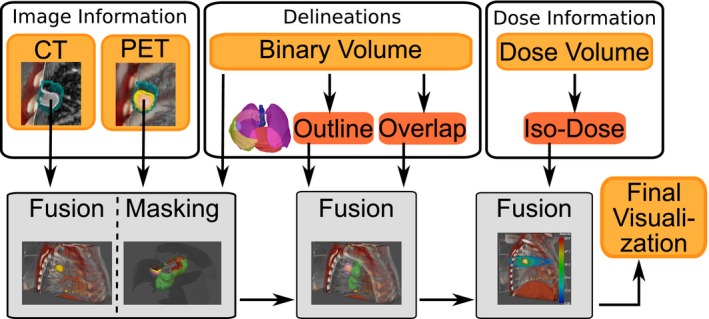
Schematic overview of image sources which are combined in the visualization system.

**Figure 2:**
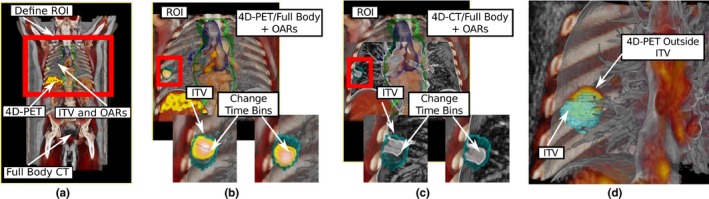
In (a), the full‐body CT is fused with 4D PET and delineations. Data sets can be clipped to a region‐of‐interest (ROI) (b). Image information can be exchanged, for instance, in (c) the 4D‐CT is used instead of the 4D‐PET. A slider can be used to navigate time bins [see (b) and (c)] and access all image information over the whole breathing cycle. Example of an ITV which does not cover the target is shown in (d).

The rendering framework supports different types of data sources and the fusion thereof: imaging data, delineations, and dose distribution. This is represented by the three blocks in Fig. 1. Representative data for one patient as used in 4D‐VS for the three clinical tasks T.1–T.3, can be found in Table 1. Data sets involved have different sizes, spatial resolutions and cover different anatomical regions of the patient [e.g. 4D‐PET/CT covers only a subvolume of the full‐body CT as in Fig. 2(a)] and are supported without further preprocessing. For our tasks, PET and CT are used as image information. However, image data from other modalities are also supported. Target and OAR delineations are represented as binary volumes and temporal delineations are supported. Dose distribution data sets are provided as 3D volumes with values in Gray (Gy) units, and were calculated using a treatment planning system (Oncentra MasterPlan v4.3).

**Table 1:** Representative imaging sources with sizes and resolutions of one patient used for the three clinical tasks

Image source	Resolution in (mm^3^)	Dimensions in (pixel)	Time bins
Full‐body CT	1.37 × 1.37 × 4	512 × 512 × 234	–
4D‐CT	1.17 × 1.17 × 2	512 × 512 × 89	10
4D‐PET	4 × 4 × 4	144 × 144 × 45	10
Planning CT	0.97 × 0.97 × 3	512 × 512 × 112	–
Delineation	0.97 × 0.97 × 3	≤ planning CT	≤ 10
Dose volume	5 × 5 × 3	90 × 61 × 112	–

### 2.B Multimodal rendering core

All volume visualizations take advantage of GPU acceleration, and are, for the main part, implemented using CUDA.^19^ Each type of data source gets handled in a slightly different way, and will in the end be combined by fusing the different data sources into a final visualization (see Fig. 1). The rendering framework organizes data sets in a unified coordinate system in GPU memory which takes into account mixed spatial resolutions and transformations between data sets in all rendering algorithms (requirement 5). 4D‐VS uses direct volume rendering and fusion^20^ of mixed resolution volume data sets for volume visualization (requirement 1). The rendering is based on a GPU accelerated ray‐casting^21^ algorithm, which uses the different data sources described above at discrete sample points during the evaluation the volume rendering integral.^20^

### 2.C Multimodal data fusion

For the fusion of image information, an accumulation level intermixing technique^22^ (color fusion) is used. Each sample point in the ray‐casting algorithm is a weighted linear combination of color and opacity values of the selected images (see Fig. 2). The weight of the linear combination can be modified via a slider allowing for adjusting the blending between volumes. The color and opacity values are defined per image source by means of a transfer function.^20^ For 4D data sets, the time bin can be changed via a slider in the user interface to select which phase of the breathing cycle should be visualized [see Figs. 2(b) and 2(c)]. Example visualizations of the fusion are depicted in Fig. 2, where a full‐body CT is fused with 4D‐PET [see Figs. 2(a) and 2(b)]. Image data can be easily exchanged during the rendering. For instance in Fig. 2(c), the 4D‐PET was exchanged with 4D‐CT, whereas all other parameters including the time bin, clipping and user‐defined rotations will be kept unchanged. This makes it possible to use multiple image information by simply exchanging them. This implements requirement 5 and parts of 6.

### 2.D Visualization and fusion of delineations

**Figure 3:**
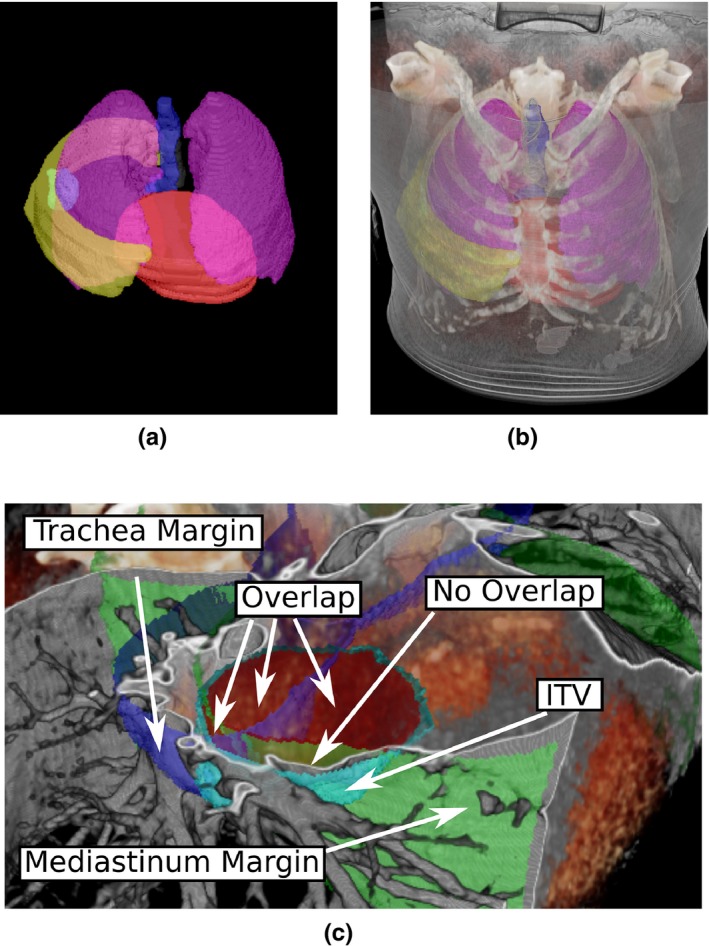
Visualization in 4D‐VS of binary volumes and fusion with image information are depicted in (a)–(b). Additional features for supporting classification of tumor localization are shown in (c).

Jointly visualizing delineations and image information (requirement 2) is implemented by visualizing binary volumes using iso‐surface rendering [see Fig. 3(a)]. During iso‐surface rendering, we determine their surface position, which is used in the ray‐casting algorithm for fusion with the volume information [see Fig. 3(b)]. Color and opacity values can be assigned to each binary volume individually, and modified in the user interface. Determining the surface position preserves the correct depth when combining delineations with volume information using the resulting color of the iso‐surface rendering (accumulation level with exclusive opacity^22^). For better depth perception, an adapted Blinn‐Phong model is used for shading^20^, ^23^ during iso‐surface rendering.

### 2.E Visualization of dose distributions

**Figure 4:**
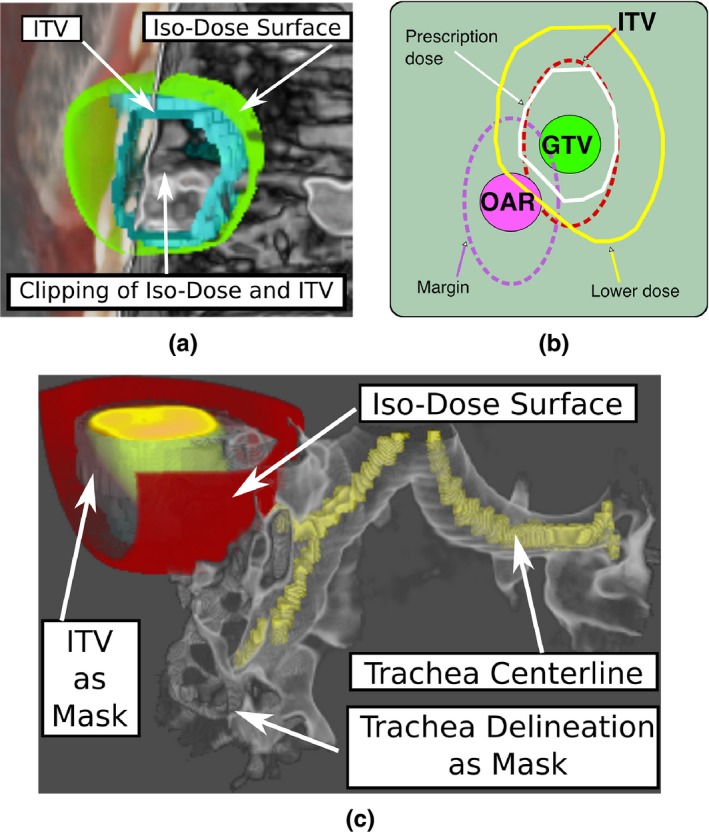
Example of the dose distribution visualization using 4D‐VS: (a) the 37.5 Gy iso‐dose surface can be evaluated against the planning ITV. A possible configuration of a target close to an OAR is shown in (b), and a combination of masking, clipping and iso‐dose rendering of a lower dose surface is shown in (c).

For implementing requirement 3, we use iso‐surface rendering^20^ for defined iso‐values given in Gray (Gy) units. Multiple values can be set in the user interface to define more than one surface. Similar to binary volumes, we use fusion of the respective surface color (accumulation level intermixing with exclusive opacity^22^) to jointly visualize iso‐dose surfaces with volume information and delineations [see Fig. 4(a)]. Additionally, the DVH for contours loaded within 4D‐VS is visualized in a separate window to complement the anatomical views, and is also necessary for task T.3.

### 2.F Volume masking using delineation information

Binary volumes can further be used for volume masking (similar to clip objects^24^) which partly implements requirement 4. Thereby, the binary volume defines a ROI and can be used to enable or disable certain volume parts (similar approach as in Ref. 6). In Fig. 4(c), only the target and an OAR (trachea) is visualized by using their delineation information as a mask. The user can decide which information should be visible inside the mask. In the example, only CT information is used for the trachea, whereas PET and CT are used inside the ITV. Furthermore, surface rendering of the delineation can be disabled. This was designed for cases in which the target is close to an OAR [see Fig. 4(b)]. Due to the constrained optimization during dose calculation, the resulting distribution might differ from the expected distribution during the prescription phase. In these cases, it might be necessary to evaluate the resulting distribution and its spatial configuration more carefully against the target or (a lower dose region) against an OAR [see Fig. 4(c)].

### 2.G Volume clipping and user interaction

**Figure 5:**
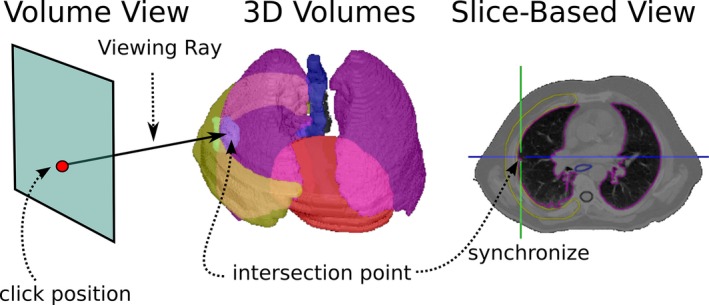
The schematic overview of binary volume picking as implemented in the visualization system.

For completing requirement 4 and 6, we implemented interactive clipping of volumes, which can be seen as a user‐defined, global ROI. In 4D‐VS, the visible part of all volumes is defined by a reference volume (usually the planning CT). Other volumes are clipped to the bounding box of the reference volume and visualized only if they are inside the reference. The bounding box of the reference volume can be interactively modified to define a smaller ROI (within the reference volume) to which all data sets are clipped. Users can define the ROI by using two sliders for each of the three coordinate axes. Each slider moves one of the planes which define the bounding box of the reference volume. In Fig. 2(a), the CT defines the reference volume and can be modified to show only a smaller part of the volume [see Figs. 2(b) and 2(c)]. This was designed to remove occluding volume information or iso‐surfaces which are not relevant for a certain task. This type of clipping by a global ROI applies for volume information, delineations and dose distributions and can be combined with volume masking [see Figs. 4(a) and 4(c)]. For target and OAR delineations, we implemented interactive point picking (see Fig. 5) to support the navigation from the 3D visualization to the 2D views (requirement 7). The closest surface point along the view direction from the 2D mouse position can be selected, and the 2D views will be re‐arranged to show the position of the surface point. This was designed for task T.1, when additional information from the slice‐based visualization is needed to assess the quality or the delineation should be modified after a region is identified where the target is not fully covered [see Fig. 2(d)].

### 2.H Volume intersection highlighting

The idea behind requirement 8 is that the classification of tumor location (see task T.2) can be determined by distances of the target to bronchial tree and mediastinum (see Ref. 25). Binary volumes for bronchial tree and mediastinum were determined automatically with the approach of,^26^ and expanded with margins defined in Ref. 25. We use these margin volumes as additional information, however since they are automatically defined, visual assessment is still required. We include these margin volumes in a separate rendering mode which highlights the intersection volume of the ITV with either one of the margin volumes [see Fig. 3(c)] for task T.2.

## 3 EVALUATION

A user evaluation was conducted for investigating the potential benefit of using the 4D visualization features as implemented in 4D‐VS by performing the three previously defined tasks T.1–T.3. For comparison to 4D‐VS, Oncentra MasterPlan (v4.3, Nucletron BV, Veenendaal, the Netherlands) was used in our evaluation, and will be denoted C‐TPS for the remainder of this article. C‐TPS is software currently used in clinical routine including the tasks T.1–T.3.

As mentioned before, our tasks are motivated by patients with malignant pulmonary lesions who are scheduled for and/or treated by stereotactic body radiation therapy (SBRT). Having very precise delineations (task T.1), is very important when using SBRT due to the high doses involved. We reduced task T.1 to a verification task only for the following reasons. First of all, we wanted the test users to concentrate on the visualizations and not on the contouring. Additionally, it reduced the time our test users needed to invest. Furthermore, if the target is delineated using two extreme phases only, quality assessment for the remaining time bins is an equally relevant task to the delineation itself, and is also relevant when using, for instance, an automated 4D segmentation algorithm.

The classification of tumor location (T.2) is relevant to decide whether or not the patient should be treated with SBRT or receive conventional treatment. In this way, T.2 is a follow‐up task on T.1.

After the dose calculation, the treatment plan will be verified and as a subtask of the verification also the dose distribution (T.3). The assessment of dose distribution T.3 is not specific to SBRT, but due the high doses involved, visualization techniques other than using the dose volume histograms (DVH) can be of interest in complicated cases, where the target is spatially close to an OAR. For this task, users were explicitly asked to take the extended volume visualization features into account in addition to the DVHs. Furthermore, we were interested in whether taking the spatial configuration into account was considered helpful by our test users. Task T.3 is a follow‐up task on T.2 and on T.1.

All three tasks have in common, that they use a visual approach for verification.

### 3.A Patient data and ground truth

Eighteen patient cases with malignant pulmonary lesions scheduled for SBRT were selected for testing of task T.1 and T.2. For reducing observer bias, we divided them in two groups (one group for 4D‐VS and one for C‐TPS), whereas each group consists of five central and four peripheral cases. As image information, we provided a full‐body CT and a 4D‐PET/CT (see Table 1).

For task T.1, we presented two ITVs for each patient which results in 18 separate test cases per patient group and system respectively. ITV_1_ was generated by a majority vote (3/4) algorithm using manual delineations of four physicians in the context of a contouring exercise. The information used for contouring consisted of 4D‐PET/CT, ungated CT and ungated PET. ITV_2_ was generated based on PET information only using a 4D‐PET segmentation algorithm.^27^ The planning ITV used for treatment of the patients was used as ground truth for estimating the quality of ITV_1_ and ITV_2_.

The conversion from DICOM‐RTSS to binary volumes was done by rasterization of each planar contour with the slice resolution of the planning CT (see Table 1). The axial resolution of binary volumes was the same as the axial planar contour distances since they were generated on the planning CT. Afterwards, we reduced the size of the binary volume by keeping the minimal part of the volume which represents the actual segmentation information to reduce the memory consumption.

The quality of ITV_1_ and ITV_2_ was determined by calculating the dice coefficient (DC) and the (average/maximum/95%) Hausdorff distances (HD) with the planning ITV. We tried to have a similar distribution of ITV quality for the two test groups by using the DC as an indicator. For 4D‐VS (first group), ITV_1_ had an average DC of 0.76 (± 0.09 SD) and ITV_2_ had an average DC of 0.5 (± 0.19 SD). The combined test data set (ITV_1_ and ITV_2_) for 4D‐VS had an average DC of 0.63 (± 0.2 SD). Respectively for C‐TPS (second group), ITV_1_ had an average DC of 0.74 (± 0.14 SD) and ITV_2_ had an average DC of 0.59 (± 0.11 SD). The combined test data set (ITV_1_ and ITV_2_) for C‐TPS had an average DC of 0.67 (± 0.14 SD).

For classification of tumor location (task T.2), the same data sets were used, however, we additionally provided margin volumes for bronchial tree and mediastinum which were determined automatically with the approach of^26^ (see above) for using volume intersection highlighting. A ground truth for tumor location was determined by an experienced radiation oncologist different from the test users. Classification was done according to the rules stated in Ref. 25 using distance measuring tools.

For task T.3, we selected only patients treated with SBRT, resulting in eight out of the 18 which were only considered for SBRT. Data sets were again split up to reduce bias (four patients for 4D‐VS and for C‐TPS). As image information, we provided the planning CT and all relevant delineations (see Table 1) of the target (planning ITV was used) and OARs. For all SBRT plans, the 3D dose distribution was calculated with Oncentra MasterPlan.

### 3.B User evaluation

Two experienced radiation oncologists (denoted as U_1_ and U_2_) performed the three tasks as described in the introduction. They were asked to give a quality rating for the ITV delineation in task T.1 and for the dose distribution in task T.3. The scale of the rating was from “1” (excellent) to “5” (poor), where a rating of “3” was defined as acceptable. Additionally, they were asked whether they are certain about their decision for the current task. This is summarized in Table 2.

**Table 2:** Task description summary and quality scale

Task description	Quality scale
T.1 Assess the quality of ITV_1_/ITV_2_ and give a rating. Indicate certainty.	1–5 (Excellent–poor)
T.2 Classify the lesions into central and peripheral. Indicate certainty.	–
T.3 Assess the quality of the dose distribution and give a rating. Indicate certainty.	1–5 (Excellent−poor)

All tasks were performed with C‐TPS and 4D‐VS. After all tasks were performed, users were asked to answer survey questions (see Table 3) for each of the systems. The survey had also a general remarks section for free comments.

**Table 3:** Survey questions with answers. Answers given as (U_1_/U_2_) or “–” if not applicable

Questions		4D‐VS	C‐TPS
Q1	How well can you imagine the 4D‐configuration of the structures? Rating 1:best – 5:worst	(1/3)	–
Q2	Does the tool have all the functionality for the ITV rating?	(y/y)	(y/y)
Q3	Does the 4D‐VS help to comprehend the test cases?	(y/y)	–
Q4	Does the tool have all the functionality for central/peripheral classification?	(y/y)	(y/y)
Q5	Does the functionality of 4D‐VS help making the decision?	(y/y)	–
Q6	Does the tool have all the functionality for dose evaluation?	(n/n)	(y/y)
Q7	Does the functionality of 3D iso‐dose help making the decision?	(y/y)	–

The visualization as presented in the manuscript was developed outside of the hospital, and the two radiation oncologists were not involved in the development of the software. The design choices were made in collaboration with technical and medical contacts (different from the test users) situated within the hospital. Hence, we needed to explain the software features in a training session as they were unfamiliar with it. During this training session, we explained the features by performing the tasks T.1–T.3 on a test data set which was not part of the evaluation set. Suggestions for the usage were made depending on the task. The testing itself was then performed unsupervised by each individual tester.

A comparison of visualization features which are available in the two systems and which are relevant for tasks T.1–T.3 can be found in Table 4.

**Table 4:** Feature Comparison of 4D‐VS and C‐TPS

Features	Task	4D‐VS	C‐TPS
2D multimodality fusion	T.1, T.2, T.3	Yes	Yes
3D multimodality fusion	T.1, T.2, T.3	Yes	No
4D multimodality fusion (2D + time and 3D + time)	T.1	Yes (slider)	No
2D/3D visualization of delineations	T.1, T.2, T.3	Yes	Yes
2D/3D fusion of delineation and volume	T.1, T.2, T.3	Yes	Yes
Mask volumes with delineations in 3D	T.1	Yes	No
Highlighting intersections of delineations in 3D	T.2	Yes	No
3D visualization of iso‐dose surfaces	T.3	Yes	Yes
ROI definition (clipping) for 3D volumes	T.1, T.2, T.3	Yes	Yes (only single volume)
ROI definition (clipping) for 3D delineations	T.1, T.2, T.3	Yes	No
ROI definition (clipping) for 3D iso‐dose surfaces	T.3	Yes	No
Interactive point picking of 3D delineations	T.1, T.2	Yes	Yes
Changing fusion parameters	T.1	Yes	Yes (only 2D)

### 3.C Visualization parameter calibration

The level and window values for PET images, which were provided by the scanner, were used in 4D‐VS and C‐TPS to provide a comparable windowing^28^ for the slice views. In 4D‐VS, these values are additionally coupled with the transfer function used for 4D volume visualization. The opacity value range was set depending on these values. Below the lower window value, PET information is transparent, and above the upper window value it has a constant opacity of 0.7. For CT images, the windowing used in the slice‐wise views could be adjusted freely in both 4D‐VS and C‐TPS, to ensure optimal parameters for the visibility (transparency) of certain tissue types. Additionally, the CT transfer function for volume visualization could be modified in 4D‐VS.

## 4 RESULTS

The average quality rating of ITVs over all test cases is shown in Table 5 (see supporting information for results for individual cases). The combined and per user average with standard deviations (SD) were both calculated. The consensus of the rating between the users was measured by calculating a conformity index (CI), which was defined as the average of the difference in the rating between U_1_ and U_2_. The CI indicating the consistency was calculated per ITV and system over all cases, and is listed for ITV_1_ and ITV_2_ in Table 5. The ratings are more consistent between users, using 4D‐VS than using C‐TPS. Using 4D‐VS leads to lower ratings and acceptance rate for ITV_1_ compared to using C‐TPS. The automatically generated ITV_2_ received low ratings in both systems. However, the acceptance rate was even lower in 4D‐VS. The level of certainty was slightly higher in C‐TPS.

**Table 5:** Results of ITV and dose distribution ratings using different systems

System	Task	Average ratings						CI
		U_1∕2_		U_1_		U_2_		
		Avg	SD	Av	SD	Avg	SD	
4D‐VS	T.1 (ITV_1_)	4.06	1.26	3.78	1.20	4.33	1.32	0.56
	T.1 (ITV_2_)	4.78	0.55	4.67	0.71	4.89	0.33	0.22
	T.1 (ITV_1∕2_)	4.42	1.02	4.22	1.06	4.61	0.98	0.39
	T.3	2.38	0.52	2.50	0.58	2.25	0.50	0.75
C‐TPS	T.1 (ITV_1_)	2.44	1.38	1.67	1.12	3.22	1.2	1.78
	T.1 (ITV_2_)	4.44	0.98	4.00	1.22	4.89	0.33	0.89
	T.1 (ITV_1∕2_)	3.44	1.56	2.83	1.65	4.06	1.21	1.33
	T.3	3.13	0.99	3.00	0.82	3.25	1.26	0.25

We defined a rating of 3 (acceptable) as the rejection threshold for ITVs, and calculated the resulting minimum, maximum, average and standard deviations (SD) of DC and HD measurements for accepted and rejected ITVs (see Table 6).

**Table 6:** Dice and Hausdorff distance comparisons to the planning ITV for rejected and accepted ITVs

Structure comparison metric	System	Accepted ITVs				Rejected ITVs			
		Min	Max	Avg	SD	Min	Max	Avg	SD
Dice	4D‐VS	0.73	0.89	0.81	0.07	0.18	0.84	0.59	0.19
	C‐TPS	0.48	0.86	0.73	0.13	0.38	0.82	0.6	0.12
Hausdorff distance (Average)	4D‐VS	1.31	4.04	2.04	1.03	1.16	7.29	3.68	1.91
	C‐TPS	0.83	8.22	2.37	1.68	1.39	9.17	3.68	2.56
Hausdorff distance (Maximum)	4D‐VS	4	11.72	7.23	2.92	4.17	23.48	11.88	5.8
	C‐TPS	4.21	52.45	10.89	11.08	5.33	52.45	15.17	12.33
Hausdorff distance (95%)	4D‐VS	2.85	7.66	4.45	1.79	2.93	14.49	7.01	3.3
	C‐TPS	2.3	25.31	5.97	5.22	2.94	25.31	8.57	6.15

Using 4D‐VS, all patients were classified correctly, and users indicated that they are certain about their decision in all but one case. Using C‐TPS, one patient was misclassified, and for all test cases users indicated that they are certain about their decision. The average quality rating of dose distributions and the corresponding certainty rates are shown in Table 7.

**Table 7:** Acceptance and certainty rate for ITV and dose distribution assessments using different systems

System	Task	Acceptance		Certainty	
		U_1_	U_2_	U_1_	U_2_
4D‐VS	T.1 (ITV_1_)	0.33	0.22	0.78	0.78
	T.1 (ITV_2_)	0.11	0	0.78	0.78
	T.3	–	–	0.5	1.0
C‐TPS	T.1 (ITV_1_)	0.88	0.67	1.0	1.0
	T.1 (ITV_2_)	0.44	0	0.78	0.89
	T.3	–	–	1.0	0.5

The overall questions and answers are listed in Table 3. The average rating for tempo‐spatial comprehensibility of 4D‐VS was 2. The feature completeness for ITV assessment and classification of tumor localization was indicated as present in both systems, however not for dose distribution assessment (Q6) in 4D‐VS. The additional functionality of 4D‐VS was indicated as helpful for all three tasks.

## 5 DISCUSSION

In this work, we presented a 4D multimodal rendering framework with additional navigation and interaction features, 4D‐VS, for the use in radiotherapy planning. 4D‐VS was applied to three specific tasks, which were also performed using the standard tool C‐TPS to investigate possible benefits. Lower quality ITVs were more likely to be detected. Ratings were more consistent for both ITVs and dose distribution. Furthermore, the classification of tumor location had a higher accuracy using 4D‐VS.

For task T.1 (quality rating of ITVs), the planning ITV was chosen as ground truth for all DC and HD measurements due to its high quality guaranteed by institutional standards. The quality of individual ITVs used in our study was measured by the DC and HD with the planning ITV (see supporting information for measurements for each data set). They had varying quality depending on their generating source, which was either an algorithm or a majority vote (see above).

The average DC values (see Table 6) for accepted ITVs using 4D‐VS was 0.81 (± 0.07 SD) and 0.73 (± 0.13 SD) for C‐TPS. The average HD values (see Table 6) for average, maximum and 95% for accepted ITVs using 4D‐VS were 2.04 (± 1.03 SD), 7.23 (± 2.92 SD) and 4.45 (± 1.79 SD). For C‐TPS, the respective HD values were 2.37 (± 1.68 SD), 10.89 (± 11.08 SD), and 5.97 (± 5.22 SD). The smallest DC (highest HD values for average, maximum and 95%) reached for contours rated as accepted, which represents a lower bound for the quality reached in our test set, was 0.73 (4.04, 11.72 and 7.66) for 4D‐VS and 0.48 (8.22, 52.45 and 25.31) for C‐TPS respectively.

Furthermore, the CI values are lower for 4D‐VS which indicates that the ratings of the ITVs become more consistent between users when using 4D visualization. ITV_2_ received lower ratings in both systems, which was expected due to the, in general, lower DC (higher HD) with the planning ITV. Overall, the detection of low quality ITVs improved and users tend to agree more in their ratings when using 4D‐VS.

**Figure 6:**
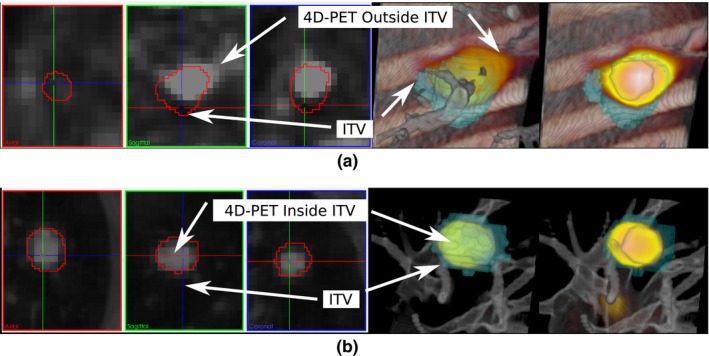
Example visualization 4D‐PET/CT and ITVs: 2D views and with 4D‐VS. Examples show a rejected ITV (a) with a DC = 0.65 and an accepted ITV (b) with a DC = 0.76.

**Figure 7:**
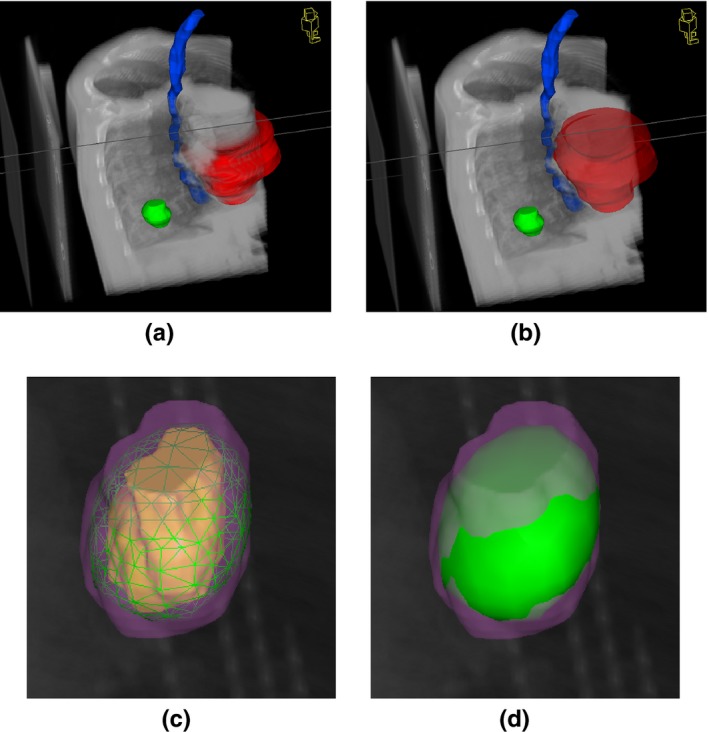
Using C‐TPS for task T.1 and T.2: Contours are visualized together with the planning CT. Clipping is applied, however, only the CT is affected. The ITV is depicted in green, the heart in red and the esophagus in blue. In (b), the heart is made slightly transparent. When compared to (a), the volume covering the heart is not shown correctly anymore. C‐TPS does not preserve the depth information of the heart when made transparent. Using C‐TPS for task T.3: Green is the 37.5 Gy iso‐dose. The planning target volume is depicted in violet, and the ITV in yellow. In (c), the iso‐dose surface is visualized as mesh, and as solid surface in (d).

Furthermore, the survey suggests that the features of 4D‐VS provide a better tempo‐spatial overview, and additional volume masking, definition of ROI and 4D multimodal visualizations are helpful for ITV assessment. An example visualization of a rejected ITV (rating = 5) is depicted in Fig. 6(a), and of an accepted ITV (rating = 2) in Fig. 6(b). The PET signal is additionally shown in the slice‐wise views on the left. In Fig. 6(a), one can clearly see that the contour does not cover the high uptake region of the PET. Although, there is the possibility to detect this by using slice‐wise views, it is more prominent in the volume visualization [see also Fig. 2(d)]. Using the additional ROI definition in 4D‐VS, volumes and contours can be clipped to define a view which “cuts” the contour open. With 4D‐VS, it is at the same time possible to navigate through all the time bins of a 4D data set, while leaving the rest unchanged. This means, by sliding through time, the breathing motion of the patient can be simulated, and the contours can be evaluated against the full 4D data set. Although, it is possible in C‐TPS to load each time bin and evaluate it, in terms of tempo‐spatial comprehensibility and time effort the 4D‐VS approach was regarded as advantageous by the users. Besides the missing temporal navigation in C‐TPS, volume visualization is available. For a better discussion of the key differences, we added example visualizations in Fig. 7showing the ITV depicted in Fig. 6(b). In C‐TPS, volume rendering is limited to a single data set, and therefore it is not possible to fuse information of PET and CT (only slice‐wise, see Table 4). The contours are only rendered at the correct spatial depth, if no transparency is applied. In Fig. 7(a), all contours are opaque, and in Fig. 7(b) the heart is partially set transparent, whereas the rest is unchanged. The heart will now be visualized on top of the volume [Fig. 7(b)] and not at its correct spatial position as in Fig. 7(a). It is possible to define ROIs in C‐TPS. However, they are only applied to volume information, and therefore it is not possible to “cut open” contours as it is in 4D‐VS (compare Fig. 6). A comparison of available features can be found in Table 4. Evaluation of the survey indicates 4D‐VS provides better spatial comprehensibility (Q1–Q3 in Table 3) and simplifies the ITV assessment. The users indicated in the survey that the ITV assessment is much faster using 4D‐VS than using C‐TPS.

For task T.2 (classification of tumor location), the differences of the two systems were less prominent, when comparing the quantitative results. All tumors were classified correctly using 4D‐VS, but only one (out of nine) patient was wrongly classified with C‐TPS. Although, the intersection highlighting was indicated as helpful for making a decision (Q5), the quantitative comparison does not show a significant improvement. In Fig. 3(c), we give an example of how 4D‐VS was used to investigate overlapping regions.

For task T.3 (quality rating of dose distribution), there is no straightforward way to define a ground truth. Therefore, we can only quantitatively compare if the ratings are below or above acceptance, and measure the CI. We observed that the average ratings of the dose distribution are slightly higher and have a slightly higher CI between users (more disagreement) using 4D‐VS than C‐TPS. This could suggest that using the additional features, presented new information which is not present in the other system and led to more disagreement. There is no clear evidence that the certainty improved, and we only observed that U_1_ was more certain when using C‐TPS, and U_2_ when using 4D‐VS. Figure 7 shows example volume visualizations with iso‐dose surfaces and contours as available in C‐TPS. Iso‐dose surfaces can be visualized as meshes or as solid surfaces. The ROI is only applied to the volume information. Figure 4(a) shows how the combination of multimodal fusion and transparent contours and iso‐dose surfaces as available in 4D‐VS can help to investigate the dose distribution. In cases where an OAR is close to the target [see Fig. 4(b)], additional volume masking can be used in 4D‐VS for showing only spatially relevant information. The OAR and the target can be masked [see Fig. 4(c)] to investigate a tumor and its dose distribution close to the trachea. Although our study gives no clear evidence that this improves the quality assurance of dose distributions, in our survey the visualization was remarked as helpful for decision making (Q7). Especially for central tumors where high precision is necessary, this may increase the spatial perception of the dose distribution, as the spatial information is not directly visible in the DVH. However, feature completeness for dose distribution assessment (Q6) using 4D‐VS was answered “no” by both users. They remarked volume overlaps should be supported by an additional display showing overlap volumes in numbers and volume to dose relationships. A noteworthy limitation is that our test data included only 3D calculated dose distributions derived from routine 3D RT‐planning. Those were combined with 4D image information, and thus the judgment would not include 4D accumulated doses but only gives a rough idea of the relation of the target to the location of the dose distribution.

Even though users were unfamiliar with 4D‐VS, after a short introduction, they established their own workflow for T.1–T.3. The good spatial overview and additionally using clipping for defining ROIs was remarked as very helpful. It was also remarked that additional training might increase the quality and could further reduce time effort.

## 6 CONCLUSION

Our proposed visualizations were generally well approved by the test users. They emphasized the helpfulness of the temporal visualization features of multimodal images and the fusion with target and OAR delineations as well as improved spatial comprehensibility. Our study also found that lower quality of ITVs are more likely to be detected when using dedicated 4D visualizations as implemented in 4D‐VS which emphasizes on volume visualization of temporal multimodal data sets. The spatial comprehensibility might also improve tasks like the classification of tumor location, which had a higher accuracy using 4D‐VS as compared to C‐TPS. Additionally, the functionality of 3D dose visualization improved the decision making about the quality of the plan. Especially for central tumors, where OARs are close to the target volumes, this might further improve visual assessment of dose distribution, since the DVH does not provide spatial context.

## CONFLICT OF INTEREST

The authors declare no conflict of interest.

## Supporting information


**Data S1.** Supplementary document with detailed statistics and additional plots.

**Video S1.** Supplementary video demonstrating features of 4D‐VS.
