# Supplementary material for: Visualization of 4D multimodal imaging data and its applications in radiotherapy planning
Source: J Appl Clin Med Phys. 2017 Oct 29;18(6):183–93. doi: 10.1002/acm2.12209 (PMC5689910; doi:10.1002/acm2.12209)
Supplement: Supplementary file 1 — Data S1. Supplementary document with detailed statistics and additional plots. [file ACM2-18-183-s001.pdf]

I. ADDITIONAL STATISTICS AND PLOTS FOR TEST CASES USED DURING TASK T.1

TABLE I. 4D-VS ITV_1

	Case 1	Case 2	Case 3	Case 4	Case 5	Case 6	Case 7	Case 8	Case 9	min	max	avg	stddev
Hausdorff Distance (Maximum)	9.99	7.03	6.19	8.29	4	7.68	7.34	12.11	6.38	4	12.11	7.67	2.33
Hausdorff Distance (Average)	3.28	1.71	1.34	2.21	1.31	1.69	1.88	4.62	2.12	1.31	4.62	2.24	1.07
Hausdorff Distance (95 percent)	5.45	3.88	3.51	5.1	2.85	4.11	4.42	7.01	4.19	2.85	7.01	4.5	1.22
Dice	0.66	0.74	0.81	0.84	0.76	0.89	0.8	0.61	0.7	0.61	0.89	0.76	0.09
Rating U1	5	4	5	3	2	2	4	5	4	2	5	3.78	1.2
Rating U2	5	5	5	5	2	2	5	5	5	2	5	4.33	1.32
Rating Combined	-	-	-	-	-	-	-	-	-	2	5	4.06	1.26

TABLE II. C-TPS ITV_1

	Case10	Case11	Case12	Case13	Case14	Case15	Case16	Case17	Case18	min	max	avg	stddev
Hausdorff Distance (Maximum)	5.33	52.45	6.95	4.21	17.73	9.92	7.34	4.78	9.42	4.21	52.45	13.13	15.3
Hausdorff Distance (Average)	1.39	8.22	1.6	1.23	3.03	3.08	1.86	0.83	1.9	0.83	8.22	2.57	2.25
Hausdorff Distance (95 percent)	3.3	25.31	3.88	2.85	8.49	6.89	4.1	2.3	5.4	2.3	25.31	6.95	7.17
Dice	0.79	0.52	0.82	0.81	0.78	0.48	0.79	0.86	0.82	0.48	0.86	0.74	0.14
Rating U1	3	4	2	1	1	1	1	1	1	1	4	1.67	1.12
Rating U2	5	3	4	2	3	5	3	2	2	2	5	3.22	1.2
Rating Combined	-	-	-	-	-	-	-	-	-	1	5	2.44	1.38

TABLE III. 4D-VS ITV_2

	Case19	Case20	Case21	Case22	Case23	Case24	Case25	Case26	Case27	min	max	avg	stddev
Hausdorff Distance (Maximum)	17.68	16.66	21.46	23.48	4.17	11.72	13.51	14.53	7.61	4.17	23.48	14.54	6.19
Hausdorff Distance (Average)	6.44	3.91	6.29	4.98	1.16	4.04	3.25	7.29	3.74	1.16	7.29	4.57	1.9
Hausdorff Distance (95 percent)	10.25	7.99	11.88	14.49	2.93	7.66	7.09	9.8	5.83	2.93	14.49	8.66	3.41
Dice	0.33	0.46	0.18	0.63	0.72	0.73	0.65	0.37	0.45	0.18	0.73	0.5	0.19
Rating U1	5	5	5	5	4	3	5	5	5	3	5	4.67	0.71
Rating U2	5	5	5	5	5	4	5	5	5	4	5	4.89	0.33
Rating Combined	-	-	-	-	-	-	-	-	-	3	5	4.78	0.55

TABLE IV. C-TPS ITV_2

	Case28	Case29	Case30	Case31	Case32	Case33	Case34	Case35	Case36	min	max	avg	stddev
Hausdorff Distance (Maximum)	9.13	17.18	9.27	5.86	31.8	9.27	9.24	6.62	18.04	5.86	31.8	12.93	8.25
Hausdorff Distance (Average)	2.2	3.85	3.4	1.46	9.17	2.87	3.07	1.42	3.88	1.42	9.17	3.48	2.32
Hausdorff Distance (95 percent)	5.65	8.71	6.35	3.76	18.48	6.61	5.9	2.94	9.95	2.94	18.48	7.59	4.62
Dice	0.6	0.56	0.59	0.71	0.38	0.52	0.61	0.76	0.61	0.38	0.76	0.59	0.11
Rating U1	5	5	3	5	5	3	3	2	5	2	5	4	1.22
Rating U2	5	5	5	5	5	5	5	4	5	4	5	4.89	0.33
Rating Combined	-	-	-	-	-	-	-	-	-	2	5	4.44	0.98

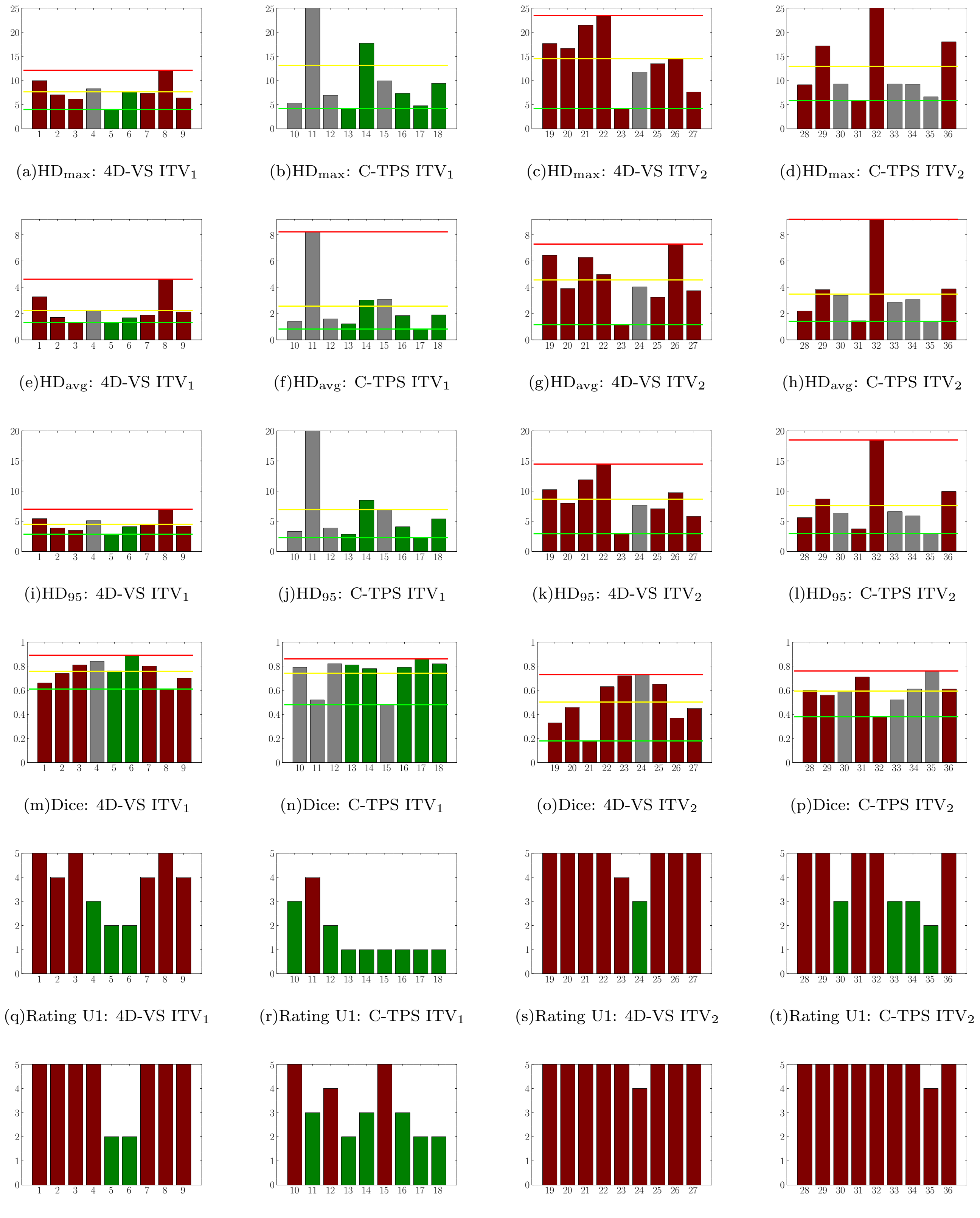

(a) HD_{max}: 4D-VS ITV_1 (b) HD_{max}: C-TPS ITV_1 (c) HD_{max}: 4D-VS ITV_2 (d) HD_{max}: C-TPS ITV_2

(e) HD_{avg}: 4D-VS ITV_1 (f) HD_{avg}: C-TPS ITV_1 (g) HD_{avg}: 4D-VS ITV_2 (h) HD_{avg}: C-TPS ITV_2

(i) HD_{95}: 4D-VS ITV_1 (j) HD_{95}: C-TPS ITV_1 (k) HD_{95}: 4D-VS ITV_2 (l) HD_{95}: C-TPS ITV_2

(m) Dice: 4D-VS ITV_1 (n) Dice: C-TPS ITV_1 (o) Dice: 4D-VS ITV_2 (p) Dice: C-TPS ITV_2

(q) Rating U1: 4D-VS ITV_1 (r) Rating U1: C-TPS ITV_1 (s) Rating U1: 4D-VS ITV_2 (t) Rating U1: C-TPS ITV_2

(u) Rating U2: 4D-VS ITV_1 (v) Rating U2: C-TPS ITV_1 (w) Rating U2: 4D-VS ITV_2 (x) Rating U2: C-TPS ITV_2

FIG. 1. Graphical overview of individual test cases used in task T.1. Columns represent results for 4D-VS ITV_1, C-TPS ITV_1, 4D-VS ITV_2 and C-TPS ITV_2 (in that order). The first four rows represent the quality measures used to compare ITVs to the planning ITV. From row one to row four these measures are: maximum (HD_{max}), average (HD_{avg}), 95% (HD_{95}) Hausdorff distance and the dice coefficient. They also have lines for the minimum (green), average (yellow) and maximum (red) values within one subplot. For plots where the maximum value lines were omitted, the maximum value was cropped to the highest displayed y-coordinate (see previous tables for the exact value). The last two rows represent the user ratings for U1 and U2. The bar colors were chosen as follows. Green bar represents an accepted case, a red bar represents a rejected case and a gray bar (does not apply to user ratings) represents a case which was rejected by one user and accepted by the other.
